# A Rare Case of Intravascular Large B-cell Lymphoma Presenting as Fever of Unknown Origin With Unexplained Proteinuria

**DOI:** 10.7759/cureus.76718

**Published:** 2025-01-01

**Authors:** Dharam P Bansal, Ram K Jat, Suchita Verma, Medha Gupta, Shraddha Mehta

**Affiliations:** 1 Department of General Medicine, Mahatma Gandhi Medical College and Hospital, Jaipur, IND; 2 Department of General Medicine, Mahatma Gandhi Medical College and Hospital, Jaipur, Jaipur, IND; 3 Department of Pathology, Unipath Speciality Lab, Ahmedabad, IND

**Keywords:** fever of unknown origin, hemophagocytic lymphohistiocytosis, intravascular large b-cell lymphoma, ivlbcl, lymphoma, proteinuria, renal biopsy

## Abstract

Intravascular large B-cell lymphoma (IVLBCL) is a subtype of extranodal diffuse large B-cell lymphoma, characterized by neoplastic B-cell proliferation within blood vessels. Its nonspecific presentation, often as fever of unknown origin (FUO) and systemic symptoms, makes diagnosis challenging. We report the case of a 66-year-old male patient presenting with persistent fever, weight loss, and respiratory failure. Initial investigations, including bone marrow biopsy and imaging, were inconclusive. The lack of response to empirical therapy and the patient’s worsening condition presented us with a diagnostic dilemma. The unexplained proteinuria led us to perform a renal biopsy, which confirmed IVLBCL through the identification of atypical B-cells in glomerular capillaries. This case highlights the importance of considering IVLBCL in persistent FUO cases and the role of specific tissue biopsy in diagnosis, even with nonspecific bone marrow studies. Early detection and prompt treatment are crucial, but prognosis remains poor due to diagnostic delays.

## Introduction

Intravascular large B-cell lymphoma (IVLBCL) is a rare and aggressive subtype of extranodal diffuse large B-cell lymphoma characterized by the proliferation of neoplastic B-cells predominantly within the lumens of blood vessels, particularly capillaries [1]. This distinct localization contributes to its unique clinical presentation and challenges in diagnosis. Three types have been described as classical (Western variant), cutaneous, and hemophagocytic lymphohistiocytosis (HLH) associated (Asian variant) [2].

Intravascular large B-cell lymphoma often presents with nonspecific signs and symptoms that vary depending on the organs affected. Common symptoms include fever of unknown origin (FUO), neurological deficits, and cutaneous manifestations. Due to its intravascular nature, IVLBCL typically does not present with significant lymphadenopathy or a detectable mass, making imaging studies like computed tomography (CT) scans less effective in diagnosing the disease. Fever of unknown origin is often one of the first signs of IVLBCL (87%-100%), prompting further investigation [3]. The fever is typically unresponsive to antibiotics, leading to a prolonged and often frustrating diagnostic process.

The prognosis of IVLBCL has historically been poor due to its aggressive nature and the challenges in early detection. Most cases are diagnosed post-mortem on autopsy [4]. However, the overall survival rates have improved with the advent of immunochemotherapy.

## Case presentation

We present a 66-year-old hypertensive male patient, a smoker, and resident of Rajasthan who came to our institution in March 2023 with the chief complaints of low-grade fever for three months; dry cough, significant weight loss, and generalized malaise for two months; and black-colored stool for two weeks. On general examination, he had pallor, pedal edema, an axillary temperature of 100°F (febrile), and oxygen saturation of 88% on room air. The patient had bilateral infrascapular crepitations with scattered rhonchi on auscultatory examination.

Previously, the patient had been evaluated in a premier institute in India for a fever of unknown origin, where bone marrow studies and whole-body positron emission tomography (PET) scans revealed no significant abnormalities except mild splenomegaly, and serum protein electrophoresis suggested polyclonal gammopathy. The patient was started on empirical weight-based anti-tubercular therapy (isoniazid, rifampicin, pyrazinamide, and ethambutol (HRZE)). On admission, the arterial blood gases were suggestive of type-one respiratory failure with hemoglobin of 5.3 mg/dL, and brain natriuretic peptide (BNP) was raised (198 pg/mL). We made a provisional diagnosis of anemia of chronic disease, with acute exacerbation of chronic obstructive airway disease, and started the patient on intravenous ceftriaxone (2 g) and doxycycline (200 mg), continued oral anti-tubercular therapy (HRZE), inhalational bronchodilators, and steroids, and transfused one unit of packed red cells while supplementing oxygen support via nasal prongs.

Lab investigations on day one revealed that the patient had newly diagnosed hypothyroidism (subclinical). He also had microcytic anemia with thrombocytopenia with no atypical cells on peripheral smear; low serum iron, total iron binding capacity; raised inflammatory markers (erythrocyte sedimentation rate, C-reactive protein, procalcitonin, lactate dehydrogenase, triglycerides, ferritin, and fibrinogen levels); and normal coagulation parameters and proteinuria (Tables 1-2). While investigations were sent, the patient continued to have a high-grade fever (maximum of 102.4°F) with oxygen requirement, and antibiotic coverage was escalated due to no improvement in the patient's condition. In order to investigate the complaint of black stools, an upper gastrointestinal endoscopy was planned but was not done since the patient was on continuous oxygen support.

**Table 1:** Laboratory parameters from days one to seven Hb: hemoglobin; TLC: total leucocyte count; AST: aspartate aminotransferase; ALT: alanine aminotransferase; ESR: erythrocyte sedimentation rate; CRP: c-reactive protein

Day	01	03	07	Reference values
Hb (g/dL)	5.7	5.4	6.2	12-18
TLC (cells/mm^3^)	4400	5500	5200	4000-11,000
Platelet (cells ×10^3^/mm^3^)	85	110	95	150-450
Creatinine (mg/dL)	1.2	1.3	1.1	0.52-1.25
Direct bilirubin (mg/dL)	0.3	0.5		0.2-1.3
AST (IU/L)	45	47.3		15-46
ALT (IU/L)	12.9	14.2		13-69
ESR (mm/1^st^ Hour)	69	43		<15
CRP (mg/L)	56.6	38.4		<10

**Table 2:** Blood investigations RDT: rapid diagnostic test; IgM: immunoglobulin M; LDH: lactate dehydrogenase; TIBC: total iron binding capacity; TSH: thyroid-stimulating hormone; T3: triiodothyronine; PT: prothrombin; RA factor: rheumatoid factor; ANA by IFA: antinuclear antibody by immunofluorescence; ACE: angiotensin-converting enzyme; IL: interleukin; CPK: creatine phosphokinase; MPO: myeloperoxidase; ANCA: anti-neutrophil cytoplasmic antibodies; PR3: proteinase 3; PNH: paroxysmal nocturnal hemoglobinuria

Investigation	Result	Reference value
Malaria antibody RDT	Negative	--
Dengue NS1 antigen	Negative	--
Dengue IgM antibody	Negative	--
Scrub typhus IgM antibody	Negative	--
*Brucella *IgM antibody	Negative	--
*Rickettsia *IgM antibody	Negative	--
Procalcitonin (ng/mL)	0.75	Moderate risk- 0.5-2.0
LDH (U/L)	2726.3 U/L	120-246
Fibrinogen (mg/dL)	603 mg/dL	220-496
Serum total iron	28.7 μg/dL	40-180 μg/dL
TIBC	228 μg/dL	261-495 μg/dL
Ferritin (ng/mL)	569	10-210
TSH (µIU/mL)	6.125	0.465-4.68
T3 (ng/mL)	0.85	0.97-1.69
Triglyceride (mg/dL)	239	<150
PT (s)	12	12
24-hour urinary protein (mg/24 hours)	1350	42-225
Haptoglobulin (mg/dL)	85.4	30-200
RA Factor (IU/mL)	8.2	<20
ANA by IFA	Negative	--
Serum ACE	68 U/L	16-85 U/L
IL-6 (pg/mL)	3.2	<4.4
CPK (U/L)	20	30-170
C3 complement (mg/dL)	102	88-102
C4 complement (mg/dL)	35	16-48
MPO/p-ANCA (U/mL)	Negative (0.5)	<5
PR3/c-ANCA (U/mL)	Negative (2.1)	<5
PNH flow cytometry	Negative	--
Stool for occult blood	Negative	--
Stool routine examination	Not significant	--
Blood culture and sensitivity	No growth	--
Urine culture and sensitivity	No growth	--

**Figure 1:**
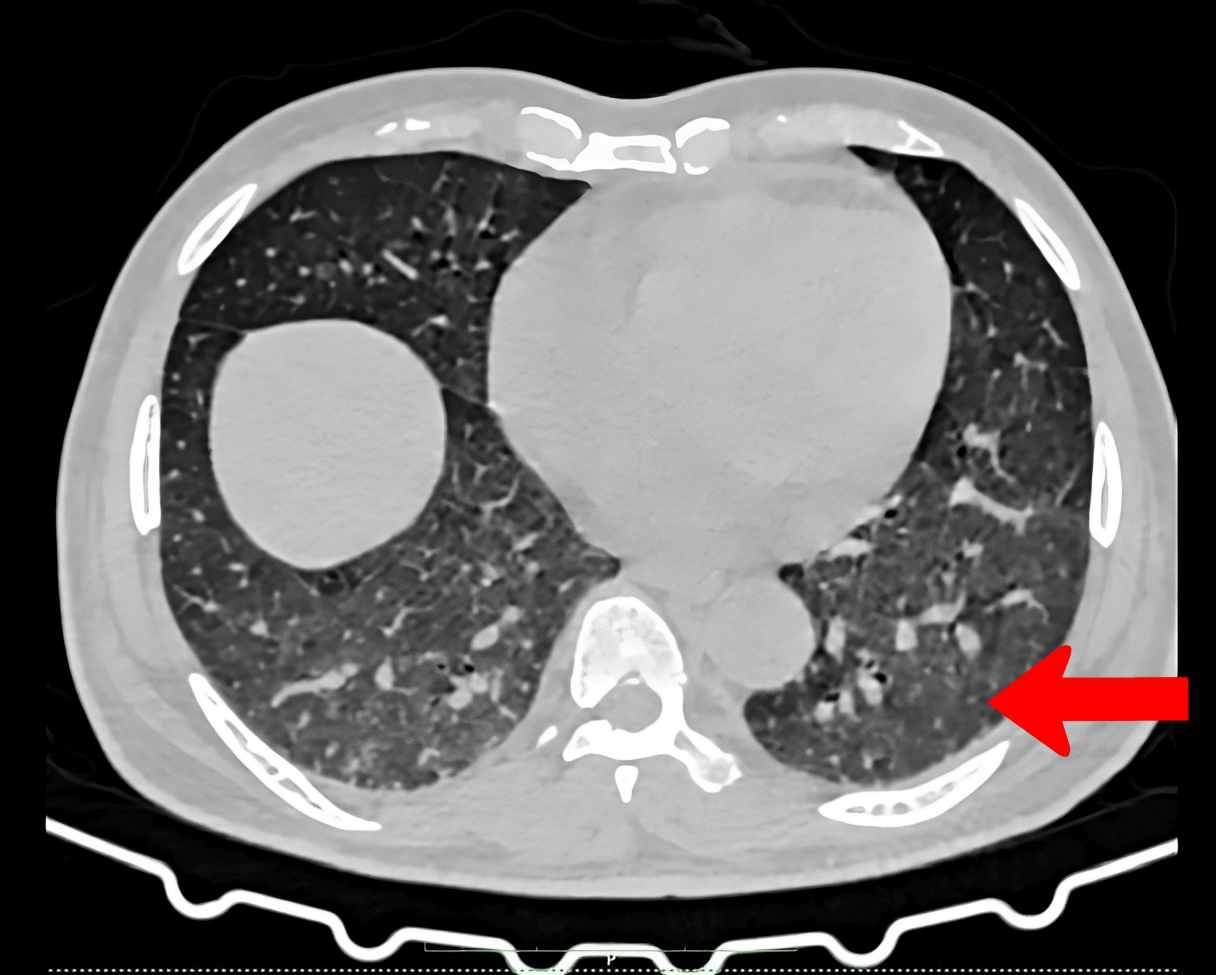
High-resolution CT images of the axial section of the thorax The red arrow shows ground-glass opacities in bilateral lung fields.

**Figure 2:**
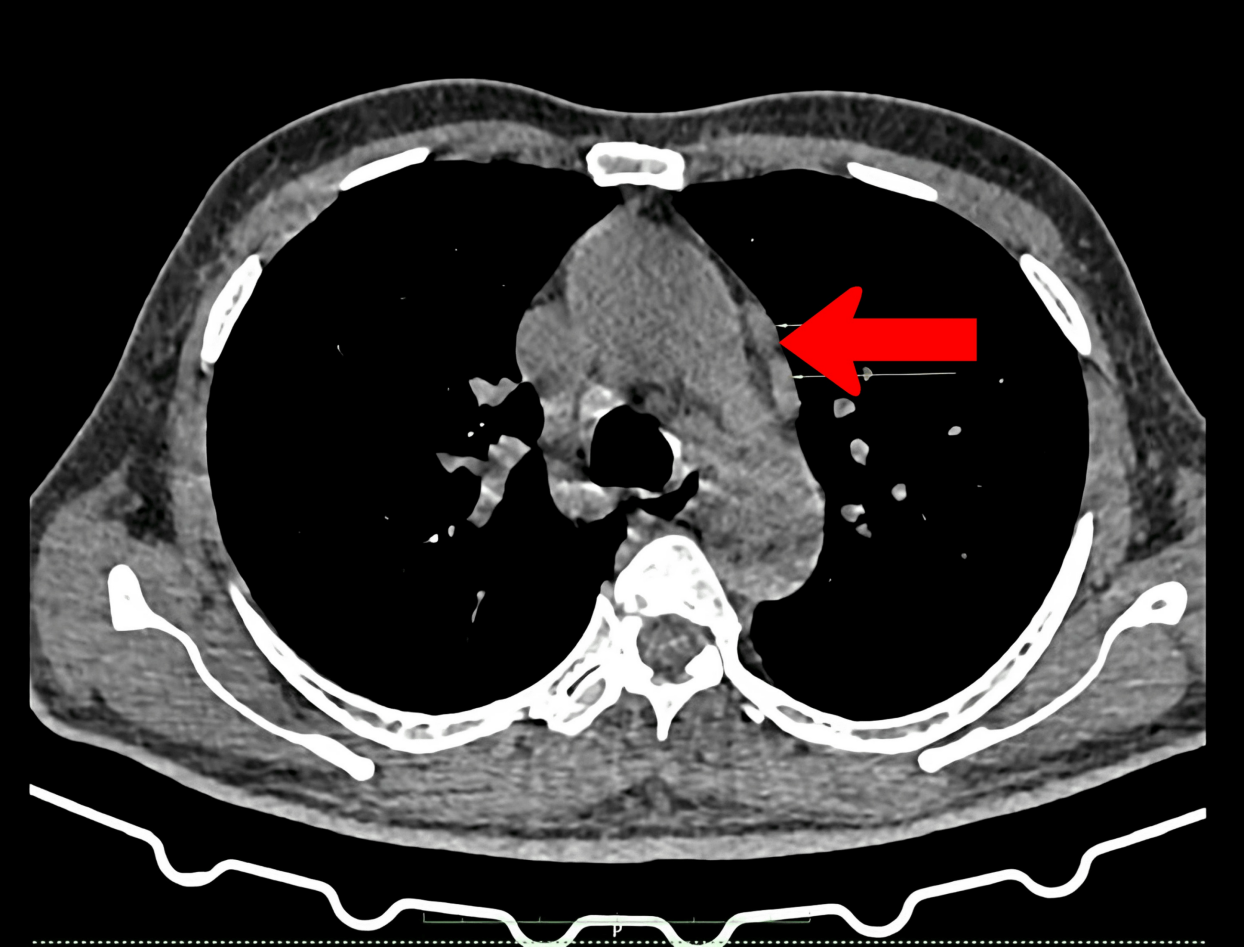
High-resolution CT images of the axial section of the thorax Red arrow points towards the largest mediastinal lymph nodes in the para-aortic window.

**Figure 3:**
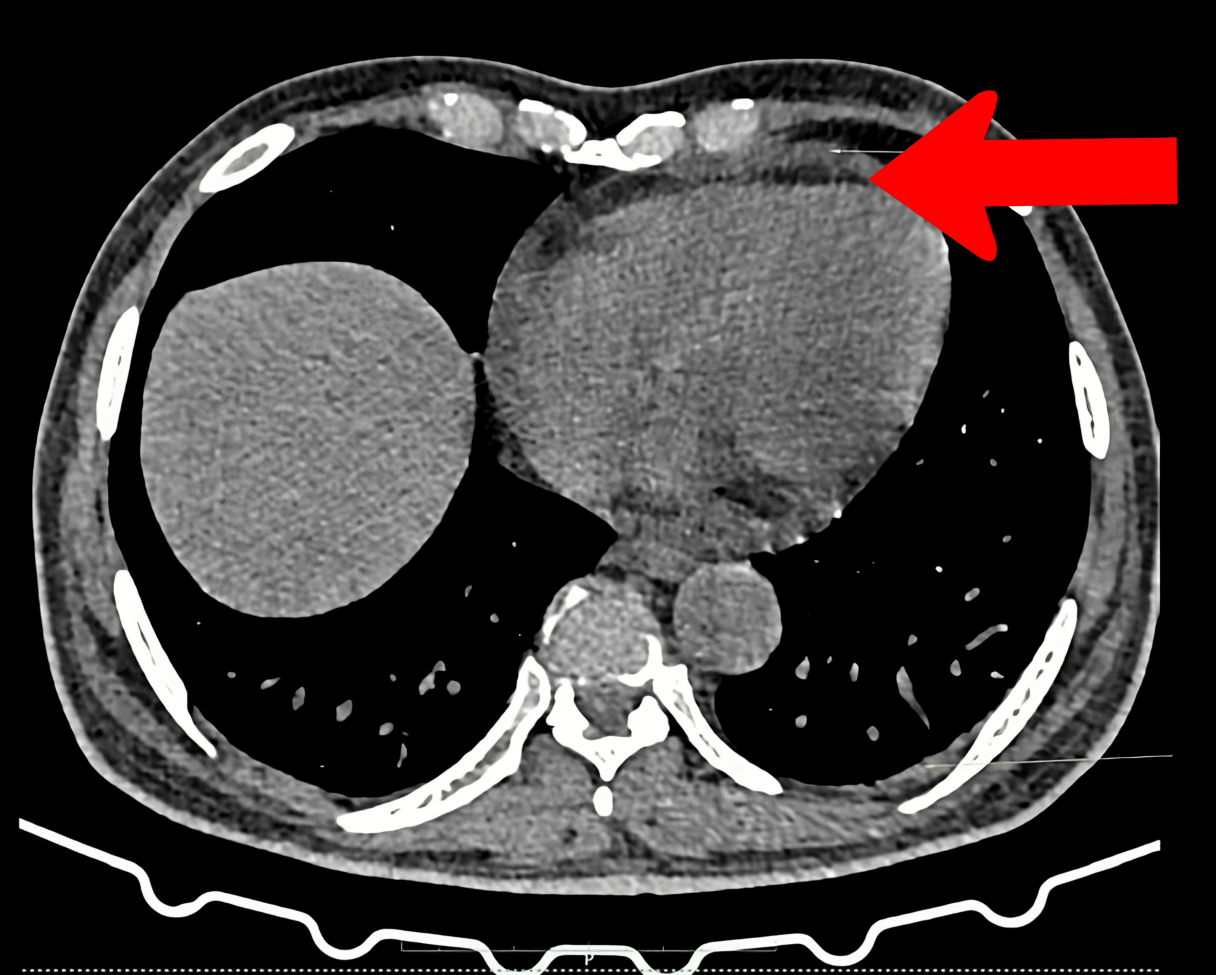
High-resolution CT images of the axial section of the thorax Red arrow shows mild pericardial effusion.

High-resolution computed tomography (HRCT) of the chest showed ground glass opacities (Figure 1), very mild bilateral pleural effusions, and the possibility of mild pulmonary edema with enlarged mediastinal lymph nodes, the largest of size 15 × 12 mm (Figure 2). There was mild pericardial effusion (Figure 3). The Mantoux test was negative, and with no clear focus on infection and a negative microbiology report, we decided to stop anti-tubercular therapy. To explore the possibilities of hemolytic anemias, vasculitis, sarcoidosis, autoimmune disease, and thrombotic microangiopathy, we proceeded to get serum haptoglobin, myeloperoxidase (MPO), proteinase 3 (PR3), serum angiotensin-converting enzyme (ACE) levels, rheumatoid arthritis (RA) factor, creatine phosphokinase, C3/C4 complement levels, antinuclear antibody by immunofluorescence, and paroxysmal nocturnal hemoglobinuria (PNH) flow cytometry, all of which were negative (Table 2). With no conclusive reports, we decided to get a repeat bone marrow study. The biopsy revealed a hypercellular marrow showing no morphological evidence of lymphomatous involvement nor hemophagocytosis. While investigations were being done, the patient had a daily high-grade fever, oxygen dependence, and increased drowsiness with no neurological deficit.

**Figure 4:**
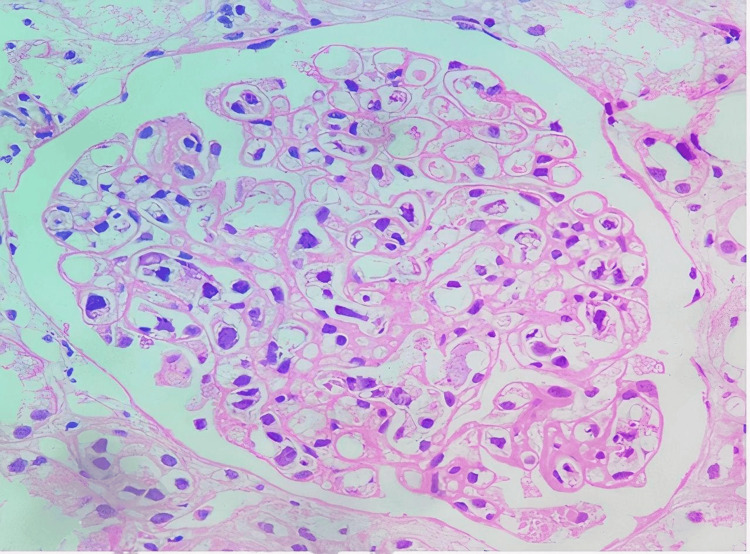
Histopathology of the renal biopsy showing hematoxylin and eosin stain of intravascular B cell lymphoma in glomerular capillaries

**Figure 5:**
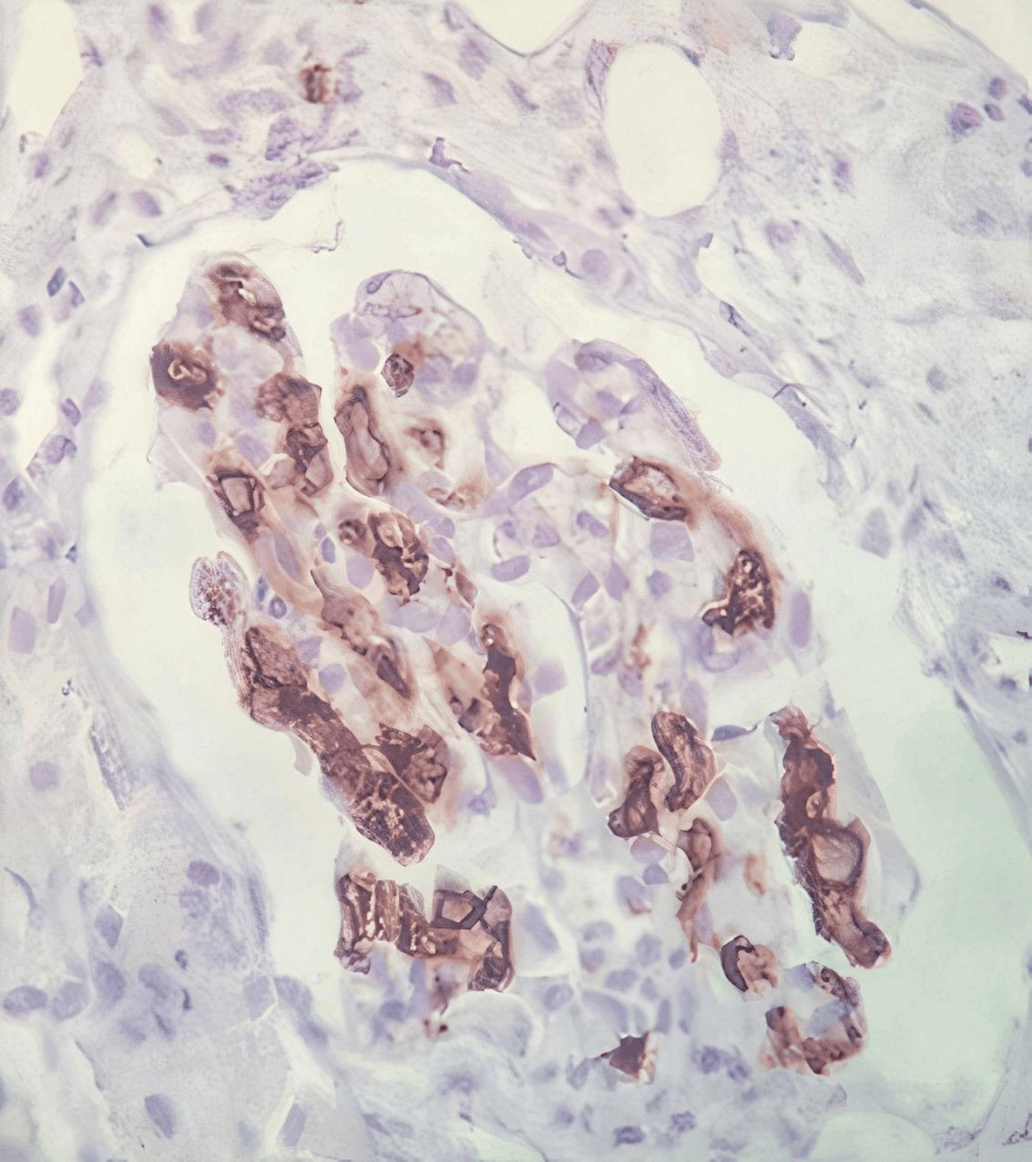
Histopathology of the renal biopsy showing CD20 immunohistochemistry

**Figure 6:**
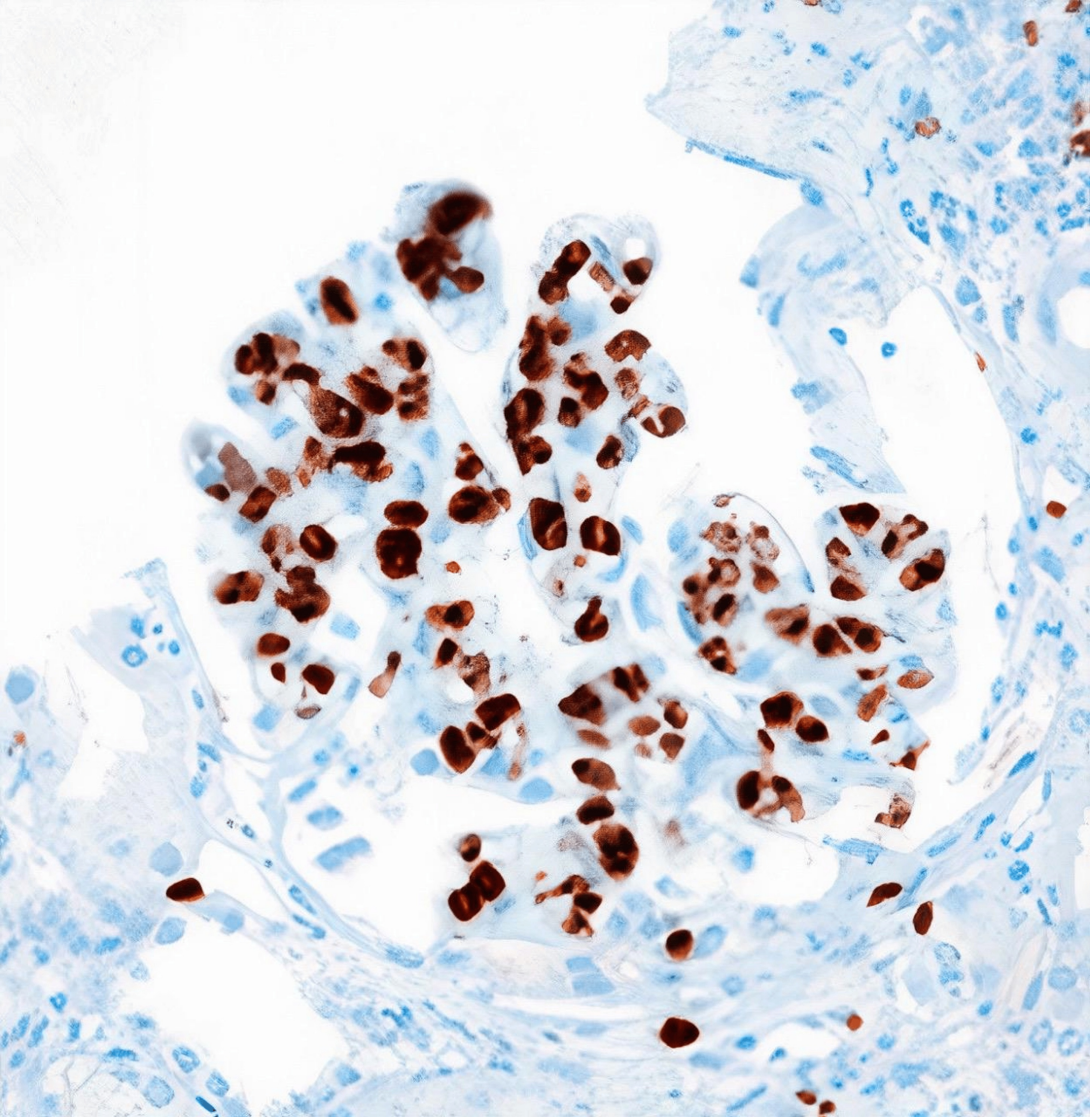
Histopathology of the renal biopsy showing PAX-5 staining reveals a Ki-67 proliferation of approximately 70%.

On day seven of admission, we had an elderly patient with diagnostic clues of fever, pancytopenia, splenomegaly, polyserositis, mediastinal lymphopathy, renal impairment, and polyclonal gammopathy. The differentials we considered were amyloidosis, Castleman’s disease, and glomerulonephropathy of unknown etiology. We faced a dilemma when choosing a biopsy site between mediastinal lymph nodes and the kidney. Ultimately the decision to get a renal biopsy was made owing to the unexplained proteinuria, high lactate dehydrogenase (LDH), and bicytopenia. The biopsy showed large, atypical cells in the glomerular capillary lumens (Figure 4); the atypical cells were positive for CD20 (Figure 5), and Ki-67 was approximately 70% (Figure 6). With this, we reached the final diagnosis of intravascular large B-cell lymphoma. To explore the possibility of added bone marrow involvement, the bone marrow study in our institution was reviewed but did not reveal any atypical cells within the microvasculature. A lymph node biopsy also could have been done to see either the infiltrative spread of the disease or reactive involvement (rare possibility) [5].

Following the diagnosis of intravascular large B-cell lymphoma, a hematology consultation was taken. The patient received a pre-phase regimen of steroids, resulting in decreased oxygen requirements and resolution of fever. Discharge was planned with instructions for hematology follow-up in two weeks to commence induction chemotherapy, but the patient was lost to follow-up. One month after discharge, the patient presented to our emergency department following circulatory collapse and succumbed to the disease.

## Discussion

Intravascular large B-cell lymphoma (IVLBCL) is rare, aggressive, and difficult to diagnose. In our case, we had a patient with symptoms of high-grade fever, weight loss, and body aches who did not respond to antibiotics and supportive therapy. We considered a multisystem, chronic infectious process (tuberculosis and *Rickettsia*), and we started empirical treatment that did not improve the patient’s condition and sent relevant serological investigations that were within normal limits. Then, we investigated autoimmune and vascular causes of fever since our patient had bicytopenia, raised LDH, hyperfibrinogenemia, and high ferritin. We systematically ruled out PNH, other autoimmune hemolysis, and sarcoidosis. The clinical criteria of the modified 2009 criteria for HLH diagnosis were fulfilled (Appendix A) [6], but we kept investigating further for secondary causes. The PET-CT scan showed mild splenomegaly, and the bone marrow study was not conclusive. This ruled out a solid organ and hematological malignancy, which made the diagnosis and treatment process even more difficult. This is consistent with other case reports where imaging was insufficient for diagnosis [7, 8]. Such diagnostic confusion is common in IVLBCL, as the disease often mimics other conditions, earning it the moniker "the great imitator" [9].

After much consideration, we decided to perform a tissue biopsy from either a mediastinal lymph node measuring 15 × 12 mm (to diagnose Castleman’s disease, disseminated tuberculosis lymphoma, or any other granulomatous disease) or the kidney (to diagnose amyloidosis, thrombotic microangiopathy, or systemic vasculitis). Lymph node infiltration is rare in IVLBCL but can be seen in advanced stages [10,11]. Ultimately, we performed a renal biopsy because of the presence of unexplained proteinuria, which was a strong diagnostic clue. The visualization of large intravascular neoplastic cells in the renal capillaries with immunohistochemical CD20 positivity and a Ki-67 index of 70% clinched the diagnosis of IVLBCL.

The World Health Organization (WHO) outlines three variants of IVLBCL: classical subtype, cutaneous subtype, and hemophagocytic subtype [1]. The classical variant often presents with symptoms of fever of unknown origin, neurological symptoms, and specific organ symptoms. Peripheral blood films are rarely diagnostic due to the intramural location of tumor cells. The cutaneous variant has the best prognosis and is diagnosed on skin biopsy. Lesions have classic peau d’orange, purpuric, or ulcerative appearance [4]. The hemophagocytic type has been mostly reported in Asia and has an aggressive course, often presenting with hemophagocytic syndrome, thrombocytopenia, hepatosplenomegaly, and bone marrow infiltration. Renal and lung involvement is also seen [11]. The essential diagnostic criteria outlined by the WHO include the presence of large lymphoid cells with centroblastic, immunoblastic, or anaplastic morphology that are restricted to intravascular spaces with positive pan B-cell markers [1].

Intravascular large B-cell lymphoma is commonly diagnosed on examination of vessels in skin biopsy and bone marrow studies, even with the involvement of other organs. In our case, we had a patient who had two bone marrow studies performed weeks apart, with additional review confirming the absence of intravascular infiltrates on the biopsied tissue. It is rare to diagnose IVLBCL on renal biopsy and even more so in the absence of marrow involvement. While there have been reported cases of renal IVLBCL, after a thorough review of the literature, we discovered that, to the best of our knowledge, this is the first case of renal IVLBCL of hemophagocytic type with lymphadenopathy without bone marrow involvement reported from India [12].

Often, IVLBCL cannot be diagnosed on conventional modalities of radiological imaging and peripheral blood examination because the neoplastic cells are confined to the intravascular spaces. The cells of IVLBCL lack adhesion molecules CD29 (integral beta-1) and CD54 (ICAM1) [13] and are deficient in metalloproteinases involved in transvascular migration. Thus, contributing to a neoplasm that cannot cross the blood vessel wall and evades the immune system. According to the WHO, bone marrow infiltrates may be sparse in the hemophagocytic variant of IVBCL [1], and that may have been the case in our patient.

It is the delay in diagnosis of IVLBCL that leads to poor patient outcomes [14], and it is important to have a high degree of suspicion for other pathologies in unremitting cases of FUO. To differentiate cases of FUO due to either underlying IVLBCL or other causes, Chen et al. [3] proposed two diagnostic models. They suggested the presence of peripheral edema, hypoxemia, neurological symptoms, interstitial lung abnormalities on CT, and elevated interleukin-10/IL-6 ratio, which can serve as diagnostic clues for distinguishing IVLBCL from other causes of FUO. On retrospective analysis based on model 1, which does not include interleukin levels, our patient had a high probability of IVLBCL. This proposal still requires robust external validation.

Treatment options include rituximab-based regimens, and for patients with central nervous system (CNS) involvement, methotrexate-based chemotherapy is preferred [15]. Our patient, unfortunately, deteriorated rapidly and ultimately succumbed to the disease. This outcome underscores the critical need for timely diagnosis and aggressive treatment. The standard rituximab, cyclophosphamide, doxorubicin, vincristine, and prednisone (R-CHOP) regimen has been shown to improve outcomes, but delay in diagnosis is the main barrier to receiving timely treatment.

## Conclusions

This case illustrates the diagnostic challenges posed by IVLBCL, a disease that often presents with nonspecific symptoms and evades detection through conventional diagnostic methods. The importance of maintaining a high index of suspicion in patients with persistent fever of unknown origin and systemic symptoms is crucial for early diagnosis and intervention. As demonstrated, tissue biopsy remains a key diagnostic tool in confirming IVLBCL, and early initiation of appropriate therapy is essential in improving patient outcomes. However, even with timely intervention, the prognosis remains guarded, highlighting the need for continued research and refinement of therapeutic approaches for this rare and aggressive lymphoma.

## Appendices

Appendix A 

**Table 3:** Hemophagocytic lymphohistiocytosis diagnostic criteria, 2009 Source: [6]

Modified 2009 hemophagocytic lymphohistiocytosis (HLH) criteria	

Molecular diagnosis of HLH	Or at least three of the following
Mutations in PRF1, MUC 13-4, STX11, Rab27A, LYST1, AP3B1, SAP, XIAP genes	1. Fever
	2. Splenomegaly
	3. Cytopenia affecting at least two cell lines
	4. Hepatitis
	And at least one of the following
	1. Ferritin elevation
	2. Elevated soluble CD25 (soluble IL-2 receptor)
	3. Hemophagocytosis seen on tissue biopsy
	4. Low/absent NK-cell activity
	Other supportive features (not required)
	1. Hypertriglyceridemia
	2. Hypofibrinogenemia
	3. Hyponatremia
